# The Prognostic Value of Sequential ^18^ F-FDG PET/CT Metabolic Parameters in Outcomes of Upper-Third Esophageal Squamous Cell Carcinoma Patients Treated with Definitive Chemoradiotherapy

**DOI:** 10.1055/s-0043-1774417

**Published:** 2023-09-13

**Authors:** Le Ngoc Ha, Nguyen Dinh Chau, Bui Quang Bieu, Mai Hong Son

**Affiliations:** 1 Department of Nuclear Medicine, Hospital 108, Hanoi, Vietnam; 2 Department of Radiation Oncology and Radiosurgery, Hospital 108, Hanoi, Vietnam

**Keywords:** ^18^ F-FDG PET/CT, sequential metabolic parameter, predictive value, definitive chemoradiotherapy, esophageal squamous cell carcinoma

## Abstract

**Objective** The aim of this study is to determine prognostic values of sequential ^18^ F-FDG PET/CT metabolic parameters in locally advanced esophageal squamous cell carcinoma (ESCC) patients treated with definitive chemoradiotherapy.

**Materials and Methods** Forty locally advanced ESCC patients treated with definitive chemoradiotherapy (dCRT) who received pre-treatment ^18^ F-FDG PET/CT (PET1) and 3-months post-treatment ^18^ F-FDG PET/CT (PET2) were enrolled in the prospective study. ^18^ F-FDG PET parameters of the primary tumor including maximum and mean standardized uptake values (SUVmax, SUVmean), metabolic tumor volume (MTV), and total lesion glycolysis (TLG) were calculated on PET delineated primary tumor. Using Kaplan-Meier curves to estimated overall survival (OS), progression-free survival (PFS), and local-regional control (LRC). Cox regression analysis was performed to find significant prognostic factors for survival.

**Results** With a median follow-up of 13.5 months, the 4-year OS, PFS, and LRC rates were 67.3%, 52.6%, and 53.4% respectively. Patients with MTV 2 > 5.7 had lower OS, PFS, and LRC rates than the lower MTV 2 group (p < 0.05). Univariate Cox regression analysis showed that MTV2 was a significant prognostic factor for OS, PFS, and LRC (p < 0.05).

**Conclusion** MTV parameter of sequential ^18^ F-FDG PET/CT could be used as a prognostic factor for OS, PFS, and LRC in locally advanced ESCC patients treated with dCRT.

## Introduction

Esophageal cancer is one of the most common cancers in the world. 1 Despite recent improvements in treatment modalities, overall survival (OS) remains poor. Upper-third esophageal carcinoma including cervical and upper thoracic tumor represents approximately 10% of esophageal cancer. 2 Surgery is considered a primary treatment modality for the middle and lower third esophageal tumors. 3 Meanwhile, radical surgery meets challenges in upper-third esophageal tumors due to the high risk of complications and death. Squamous cell carcinoma histologically accounts for over 90% of upper-third esophageal carcinoma that is sensitive to radio-chemotherapy. Therefore, definitive chemoradiation therapy (dCRT) is a standard treatment with improving survival in comparison with surgery or radiation therapy alone. 4 5 6 7

It has been proved that ^18^ F-flouro-2-deoxy-glucose positron emission tomography/computed tomography ( ^18^ F-FDG PET/CT) has an important role in staging, radiotherapy planning, restaging, and follow-up of esophageal cancer. 8 9 Esophageal tumors can be evaluated by endoscopy, ultrasound endoscopy, computed tomography, and ^18^ F-FDG PET/CT. 10 Metabolic changes measured by ^18^ F-FDG PET occur earlier than morphological changes. Therefore, ^18^ F-FDG PET/CT can detect malignant tumor, recurrent or metastasis diseases earlier than conventional imaging. 11 12 13

Recently, the prognostic role of ^18^ F-FDG PET/CT has been increasingly investigated. Several papers have shown that pretreatment quantitative parameters derived from ^18^ F-FDG PET/CT such as maximum standardized uptake value SUVmax, total lesion glycolysis (TLG), and metabolic tumor volume (MTV) were independent prognostic factors for treatment outcomes. 14 15 16 Besides that, sequential ^18^ F-FDG PET/CT after dCRT seems to be a more promising tool in prognosis of outcomes. Some studies proved that sequential ^18^ F-FDG PET/CT was helpful to evaluate the treatment outcomes as well as making decision to guide personalized therapy such as SUVs and tumor burden parameters. 17 18 Moreover, sequential SUVmax, TLG, and MTV were used as the primary parameter of quantitative ^18^ F-FDG PET/CT in prognosis outcome in few studies. 19 20 21 A study of Kim et al showed that relative change of SUVmean and MTV is related to local–regional recurrences and distant metastases after radiation therapy. 22 Li et al reported that sequential TLG was more reliable than MTV in prognosis for outcome after dCRT. 17 However, the actual prognostic role of sequential ^18^ F-FDG PET/CT parameters still being debated. 23

In this study, we aimed to assess the potential value of sequential SUVs parameters, MTV, and TLG in prognosis of local–regional control (LRC), progression-free survival (PFS), and OS in upper-third esophageal squamous cell carcinoma (ESCC) patients after dCRT.

## Materials and Methods

### Patients' Population

A prospective study with 60 consecutive upper-third ESCC patients registered from May 2017 to November 2021 at 108 Military Central Hospital. The inclusion criteria were (1) upper third esophageal tumor, (2) squamous cell carcinoma confirmed by endoscopic biopsy, (3) stage II or III by American Joint Committee on Cancer 7th, and (4) Eastern Cooperative Oncology Group performance status: 0 to 2, (5) age more than or equal to 18 years. The exclusion criteria were (1) other serious comorbidities, (2) previous radiation or chemotherapy, (3) abdominal lymph-node metastasis, and (4) insufficient follow-up data (20 patients). Forty patients underwent dCRT and had two ^18^ F-FDG PET/CT image series. Whole body ^18^ F-FDG PET/CT was performed within 2 weeks before dCRT, then sequential PET scan was repeated after treatment 12 weeks. The primary endpoint was OS and secondary endpoints were PFS and locoregional-free survival. This study was approved by the Institutional Review Board of Hospital 108 (No 127/QĐ-VNC). Written informed consent was obtained from all patients before registration.

### ^18^ F-FDG PET/CT Procedure

All patients underwent whole-body ^18^ F-FDG PET/CT in the Department of Nuclear Medicine, the Hospital 108. PET/CT scan was performed, using GE Discovery 710 (GE Healthcare, Milwaukee, Wisconsin, United States), according to the European Association of Nuclear Medicine guidelines, version 2.0. 24 Patients should be fasted at least 4 hours, and blood sugar was measured before injection of ^18^ F-FDG. The PET/CT study schedule was postponed when glucose level higher than 11 mmol/L (about 200 mg/dL). Afterward, the patients rested in the waiting room before intravenous injection of 2.5 MBq/kg body weight (±10%) of ^18^ F-FDG. The parameters of the low dose CT scan were as follows: 120 kVp, modulated milliampere-seconds (mAs), the helical slice thickness of 3.75 mm, and 0.5 s/rotation. PET images were reconstructed using an iterative algorithm with attenuation correction with CT.

### Quantitative ^18^ F-FDG Metabolic Assessment

^18^ F-FDG PET/CT images were evaluated by two experienced nuclear medicine physicians and the consensus was reached in each case. The volume of interest was set manually to exclude adjacent physiological ^18^ F-FDG-avid structures on attenuation-corrected PET images at the AW workstation version 4.7 (GE Healthcare, Milwaukee, Wisconsin, United States). Then, the region of interest in the esophageal lesions was assessed with reference to patient's symptoms, endoscopy, and CT imaging. The tumor volume was determined by iterative adaptive threshold segmentation provided by vendor (PETVCAR software, GE Healthcare). The iterative algorithm used a slope gradient vector algorithm which found a threshold value that separated the tumor from the background tissue by weighting the SUV max value within the bounding box by a “w” weight factor (where 0 ≤ w ≤ 1 with default value of 0.5). The tumor border was then semiautomatically contoured and MTV was obtained as tumor volume ( Supplement Fig. 1 ). SUVmax and SUVmean were defined as the maximum and mean value of SUV in MTV. TLG was calculated as SUVmean multiplied by MTV. All ^18^ F-FDG PET/CT derived parameters were computed by PETVCAR software (version 4.7, GE Healthcare, Milwaukee, Wisconsin, United States). ^18^ F-FDG-avid lesions were defined as uptake above that of mediastinal blood pool activity, or above the background activity. In case of noncomplete response, both PET scans would be co-registered and the sequential parameters were identified by semiautomatic delineation method based on the original location of primary tumor with manual adjustment of esophagitis and physiological high uptake regions. Tumor's quantitative parameters were SUVmax, SUVmean, MTV, and TLG computed by PETVCAR software (version 4.7, GE Healthcare, Milwaukee, Wisconsin, United States). 25 Derived parameters were collected on pretreatment ^18^ F-FDG PET/CT: SUVmax1, SUVmean1, MTV1, TLG1, and posttreatment: SUVmax2, SUVmean2, MTV2, TLG2 ( Supplement Fig. 2 ).

### Chemoradiation Therapy

dCRT was approved by tumor board in oncology institute of the Hospital 108. Gross tumor volumes were identified by the combination of contrast-enhanced CT simulation and ^18^ F-FDG PET/CT. Intensity-modulated radiation therapy (IMRT) with simultaneous integrated boost technique delivers a total dose of 60 Gy to the primary tumor and active lymph nodes, 50.4 Gy to regional lymph nodes in 28 fractions. Chemotherapy was administered with cisplatin 75 mg/m ^2^ day 1 plus fluorouracil (5-FU) 750 mg/m ^2^ from day 1 to 4 (weeks 1, 5, 9, 13) or paclitaxel 50 mg/m ^2^ plus carboplatin AUC2 (days 1, 8, 15, 22, 29).

### Follow-Up

Sequential ^18^ F-FDG PET/CT was assessed 3 months after completion of dCRT. Patients were followed up every 3 months with clinical examination, esophageal endoscopy, and chest-abdominal CT. After 2 years, the patients underwent follow-up every 6 months. OS was defined as the time from the start of dCRT to death of any cause or the last day of clinical follow-up. PFS was defined as the time from the beginning of dCRT to the day of disease progression or death of any cause or the last day of clinical follow-up. LRC was the length of time from the start of treatment to the day of progression or recurrence within irradiated field.

### Statistical Analysis

Commercial software packages were used for statistical analysis (SPSS v.22.0, IBM Corp). Categorical values were compared using the chi-square test or Fisher's exact test. Continuous variables were compared using paired Student's *t* -test with normal distribution or Mann–Whitney U test with abnormal distribution. 26 Estimating LRC, PFS, and OS was analyzed by using Kaplan–Meier methods. Cox regression analysis was used to determine the prognostic parameters for OS, PFS, and LRC. Mean value of FDG PET/CT parameters were used to identify cutoff values for OS, PFS, and LRC. The statistical significance was set at *p* -value less than  0.05. 27

## Results

### Patients' Characteristics

The general characteristics of patients was summarized in Table 1 . Forty consecutive patients, 100% male with mean age of 58, were included in the study. The majority of patients 34/40 (85.5%) were classified with stage III. Paclitaxel/carboplatin regimen was administered in 85% of patients. During the median follow-up of 13.5 months, 10 patients were death. Causes of death were tumor progression (7 patients), lung metastasis (2 patients), and esophageal perforation (1 patient).

**Table 1:** General characteristic of esophageal squamous cell carcinoma patients

Clinical characteristics	No. of patients ( *n* = 40)	Percent (%)
Age (mean ± SD)	58.0 ± 7.6	
**Sex** Male	40	100
**Tumor site** Cervical Thorax	12 28	30.0 70.0
**Pathology** Highly differentiated (G1) Moderately differentiated (G2) Poorly differentiated (G3) Unclassified (GX)	2 20 17 1	5.0 50.0 42.5 2.5
**T stage** T1b T2 T3	2 3 35	5.0 7.5 87.5
**N stage** N0 N1 N2 N3	4 23 11 2	10.0 57.5 27.5 5.0
**TNM stage** IIA IIB IIIA IIIB IIIC	1 5 22 10 2	2.5 12.5 55.5 25.0 5.0
**Chemotherapy regimen** Cisplatin/5-FU Paclitaxel/carboplatin	6 34	15.0 85.0
Interval time between the end of dCRT and PET2 (months)	3.3 ± 0.2	
Follow-up median (months)	13.5 (6–50)	

Abbreviations: 5-FU, fluorouracil; dCRT, definitive chemoradiation therapy; PET2, positron emission tomography2; SD, standard deviation.

### The Prognostic Value of Sequential ^18^ F-FDG PET/CT Parameters for LRC, PFS, OS

**Fig. 1:**
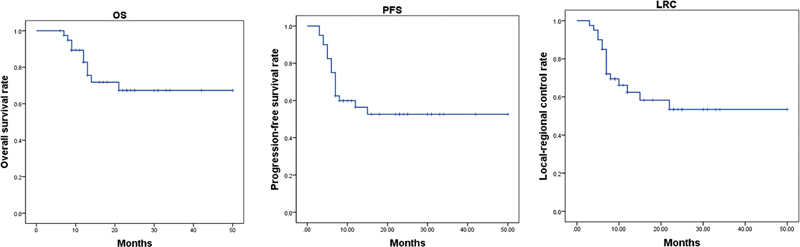
Kaplan–Meier curve for survival. LRC, locoregional recurrence; OS, overall survival; PFS, progression-free survival.

**Fig. 2:**
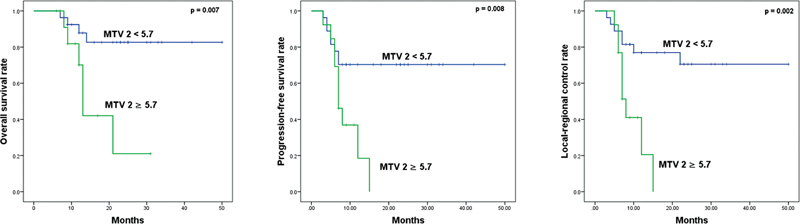
Kaplan–Meier curves for overall survival, progression-free survival, and local–regional control according to metabolic tumor volume 2 more than or equal to 5.7 and less than 5.7 mL. MTV 2, metabolic tumor volume 2.

The tumor metabolic parameters derived from PET 1 and PET 2 were showed in Table 2 . The 4-year OS, PFS, and LRC rate were 67.3, 52.6, and 53.4%, respectively ( Fig. 1 ). On Cox regression univariate analysis, MTV2 was a significant prognosis factor for OS (heart rate [HR] = 1.07, *p* = 0.022), PFS (HR = 1.05, *p* = 0.045), and LRC (HR = 1.07, *p* = 0.005) ( Table 3 ). The median OS, PFS, and LRC of patients with MTV2 of 5.7 mL or higher were 13, 7, and 8 months, respectively, which were significantly worse than that of patients with MTV2 less than 5.7 mL ( *p* < 0.05; Fig. 2 ).

**Table 2:** Changes of tumor parameters between initial and sequential ^18^ F-FDG PET/CT

	PET 1			PET 2			*p* -Value
	Mean	Median	Range	Mean	Median	Range	
SUVmax	14.5	13.6	2.9–34.1	5.5	4.0	1.6–20.9	0.000 a
SUVmean	6.7	7.0	2.3–15.1	3.1	2.9	1.3–8.7	0.000 a
MTV	18.6	13.7	0.9–55.3	5.7	1.7	0.1–32.5	0.000 a
TLG	151.3	96.3	2.2–778.4	27.4	5.0	0.2–283.8	0.000 a

Abbreviations: ^18^ F-FDG PET/CT, ^18^ F-flouro-2-deoxy-glucose positron emission tomography/computed tomography; MTV, metabolic tumor volume; SUVmax, maximum standardized uptake value; TLG, total lesion glycolysis.

a Mann–Whitney U test.

**Table 3:** Univariate Cox regression analysis for OS, PFS, and LRC in ESCC patients treated with dCRT

Parameters	OS			PFS			LRC		
	HR	95% CI	*p-* Value	HR	95% CI	*p* -Value	HR	95% CI	*p* -Value
Stage III/ II	28.37	0.31–25,959.87	0.336	3.47	0.46–26.12	0.228	1.37	0.48–3.95	0.560
Pathology G3/G1-2	2.06	0.58–7.31	0.263	1.55	0.60–4.02	0.368	0.83	0.30–2.34	0.728
Chemo CF/PC	2.79	0.72–10.86	0.138	1.68	0.55–5.12	0.358	1.33	0.38–4.68	0.655
SUVmax1	1.06	0.97–1.15	0.182	1.05	0.98–1.12	0.148	1.05	0.98–1.13	1.146
SUVmean 1	1.32	0.99–1.53	0.061	1.12	0.95–1.31	0.173	1.12	0.93–1.34	0.234
MTV 1	1.04	1.00–1.07	0.044*	1.02	0.99–1.05	0.193	1.02	0.98–1.05	0.408
TLG 1	1.00	1.00–1.01	0.060	1.00	1.00–1.01	0.237	1.00	1.00–1.01	0.322
SUVmax 2	1.12	0.98–1.27	0.094	1.08	1.0–1.18	0.070	1.12	1.02–1.22	0.013*
SUVmean 2	1.28	0.87–1.88	0.209	1.26	0.99–1.59	0.057	1.39	1.10–1.77	0.006*
MTV 2	1.07	1.01–1.13	0.022*	1.05	1.00–1.09	0.045*	1.07	1.02–1.12	0.005*
TLG 2	1.01	0.99–1.02	0.092	1.00	0.99–1.01	0.258	1.01	1.00–1.01	0.181

Abbreviations: CI, confidence interval; dCRT, definitive chemoradiation therapy; ESCC, esophageal squamous cell carcinoma; HR, heart rate; LRC, locoregional recurrence; MTV1, metabolic tumor volume1; OS, overall survival; PFS, progression-free survival; SUVmax 1, maximum standardized uptake value 1; TLG 1, total lesion glycolysis 1.

* denotes significant difference.

## Discussion

Definitive chemoradiation is the first-choice treatment of inoperable esophageal cancer, especially in patients with squamous cell carcinoma. Despite the improvement in radiotherapy techniques such as IMRT, the outcomes of ESCC patients remain poor with 5-year OS around 25 to 31%. 28 29 Tumor stage and lymph node involvement are two well-known prognostic factors of esophageal cancer. 30 31 Other clinical factors could impact on OS including malnutrition, comorbidities, and low socioeconomic status. 32 Our study showed clinical staging, tumor histological grading, and chemo regimen were not significant prognostic factors for survival ( *p* > 0.05). The primary tumor and nodal status of patients in our study could not be confirmed by pathology that might lead to inaccurate in assessment of clinical staging and histological grading. Our result is in line with previous study that showed comparable OS between carboplatin/paclitaxel and cisplatin/5-FU as dCRT in esophageal cancer patients. 33

Prognostic role of ^18^ F-FDG PET/CT has been investigated in recent studies. But which parameters derived from ^18^ F-FDG PET/CT should be used as prognostic factors for survival in esophageal cancer is still matter of debate. Our study reported that posttreatment MTV (MTV 2) was a significant factor in prognosis of LRC, PFS, and OS by univariate Cox regression analysis. Patients with MTV 2 more than 5.7 had worse OS, PFS, and LCR than that with MTV 2 of 5.7 or less ( *p* < 0.05). MTV reflects the active metabolic state of the whole tumor and it is considered to be a comprehensive parameter in prediction of treatment response and prognosis. Hence, this parameter may represent the shrinkage of the viable tumor portion after chemo and/or radiation therapy. Some studies showed that MTV was better than SUVmax that represents a small part of tumor in prognosis of esophageal cancer. 20 34 35 36 MTV2 shows the volume of metabolic lesion that remains after therapy and it may have value in prognosis of treatment response and outcome. Moreover, Tamandl et al founded that MTV2 with cutoff value of 5.8 could predict pathological complete response that correlated to OS. 37 Prognostic role of other ^18^ F-FDG PET/CT parameters was mentioned in some studies. The optimal cutoff prognostic value of SUVs, MTV, and TLG varied across studies because they may depend on histopathologic features, necrosis, heterogeneity of tumor, and methods of segmentation. 38 39 40 Li et al reported that SUVmax 2, TNM, and length of primary tumor were independent prognostic factors for OS. 17 This study used 40% of SUVmax as the lower threshold for MTV calculations, which included primary tumor and lymph node with highest SUVmax. In our study, MTV was determined by iterative adaptive threshold method that has been proved more accurate than fix threshold method. 41

MTV and TLG are emerging new parameters, and they seem to have more promising results in prognosis than other parameters. 17 19 21 22 42 The prognostic value of these parameters for treatment outcome was approved in laryngeal carcinoma and nonsmall cell lung cancer in recent studies. 43 44 A systemic review of Cremonesi et al showed that there were not constant correlation between sequential ^18^ F-FDG PET/CT parameters and clinical outcomes among studies ( Table 4 ). 45 Therefore, further research is needed with uniform protocol and method of analysis to assess prognostic value of sequential ^18^ F-FDG PET/CT.

**Table 4:** Sequential ^18^ F-FDG PET/CT parameters and clinical outcomes among studies

Author, year	No. of patients/Study style	Pathology	Protocol	Prognostic factors	Cutoff value	Outcomes
Chen et al 46 2015	34 Prospective	SCC	dCRT	MTV 1 ∆SUVmax	21 mL 70%	PFS PFS, LRC
Li et al 17 2015	160 Retrospective	SCC	dCRT	MTV 1 TLG 1 ∆SUVmax	22 mL 170 67%	OS OS OS
Kim et al 42 2016	53 Retrospective	AC	CRT + Sur	∆TLG ∆MTV ∆SUVmax	44.4% 25.5% 23.5%	OS
Li et al 21 2019	134 Retrospective	SCC	dCRT	MTV 1 TLG1 SUVmax 2 TLG 2	10.5 mL 59.8 7.8 44.3	OS OS OS OS
Kim et al 22 2019	21 Retrospective	SCC	CRT +/− Sur	∆MTV ∆SUVmean	1.14 35%	LRC DM

Abbreviations: dCRT, definitive chemoradiation therapy; DM, distant metastasis; LRC, locoregional recurrence; MTV1, metabolic tumor volume 1; OS, overall survival; PFS, progression-free survival; PFS, progression-free survival; SCC, squamous cell carcinoma; Sur, surgery; SUVmax 1, maximum standardized uptake value 1; TLG 1, total lesion glycolysis 1.

Several studies demonstrated that pretreatment ^18^ F-FDG PET/CT parameters such as SUVmax, MTV, and TLG of primary tumor were independent prognostic factors for treatment outcomes. 14 15 16 21 46 Our result showed only MTV 1 had prognostic value for OS in univariate analysis. Posttreatment residual lesion is the part of the primary tumor that is resistant to chemoradiotherapy and might directly cause recurrent or metastasis. 22 Therefore, sequential ^18^ F-FDG PET/CT might be more promising tool than pretreatment ^18^ F-FDG PET/CT in prognosis of outcomes. The prognostic role of sequential ^18^ F-FDG PET/CT in our study was in line with several studies. 21 22 42

Our study had some limitations. First, this is a single-center study that may had inherent biases. Second, metabolic parameters of sequential FDG PET/CT do not accurately reflect the effectiveness of treatment due to postradiation inflammation. In addition, only parameters of the primary tumor were evaluated, while the outcomes of ESCC actually correlated to both tumor and metastatic lymph node parameters. Moreover, the number of patient enrollment is limited and the time of follow-up is less than 5 years. Those explain why we did not find any independent prognostic factor for survival when performing multivariate Cox regression analysis in our study.

## Conclusion

Our study suggests that posttreatment MTV (MTV2) with a cutoff value of 5.7 mL can be used to prognose clinical outcomes in locally advanced ESCC patients treated with dCRT. These findings need to be validated by further studies with a larger cohort of patients.
