# Supplementary material for: The Prognostic Value of Sequential 18 F-FDG PET/CT Metabolic Parameters in Outcomes of Upper-Third Esophageal Squamous Cell Carcinoma Patients Treated with Definitive Chemoradiotherapy
Source: World J Nucl Med. 2023 Sep 13;22(3):226–33. doi: 10.1055/s-0043-1774417 (PMC10581756; doi:10.1055/s-0043-1774417)
Supplement: Supplementary file 1 — Supplementary Material [file 10-1055-s-0043-1774417-s2340007.pdf]

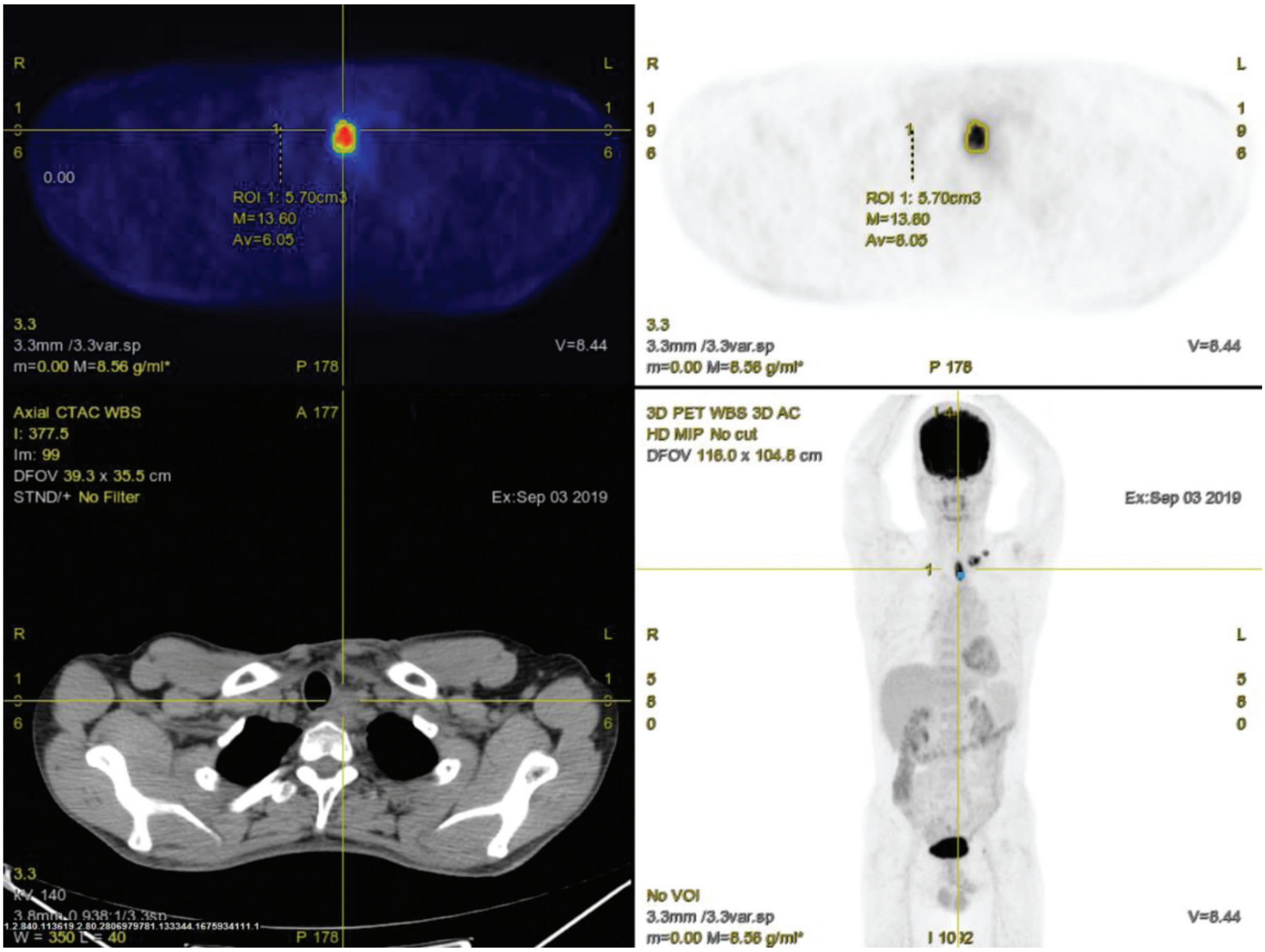


**Supplement Fig. 1** Segmentation of tumor volume. The volume of interest (VOI) was set automatically and then adjusted manually to exclude adjacent physiological $^{18}$F-FDG-avid structures on attenuation-corrected PET images at the AW workstation version 4.7 (GE Healthcare, Milwaukee, WI, USA). Then, the region of interest (ROI) in the esophageal lesions were assessed with reference to patient's symptoms, endoscopy, and CT imaging. Tumor volume was determined by iterative adaptive threshold segmentation provided by vendor (PETVCAR software, GE Healthcare). The iterative algorithm uses a slope gradient vector algorithm which finds a threshold value that separates the tumor from the background tissue by weighting the SUV maximum value within the bounding box by a 'w' weight factor (where $0 \leq w \leq 1$ with default value of 0.5). The tumor border was then automatically contoured and metabolic tumor volume (MTV) was obtained as tumor volume. SUVmax and SUVmean were defined as the maximum and mean value of SUV in the tumor volume. SUVpeak was average SUV of a 1 cm$^3$ spherical centered on the hottest point within the tumor. Total lesion glycolysis (TLG) was calculated as SUVmean multiplied by MTV. All $^{18}$F-FDG PET/CT derived parameters were computed by PETVCAR software (version 4.7, GE Healthcare, Milwaukee, WI, USA).

 © 2023. The Author(s).

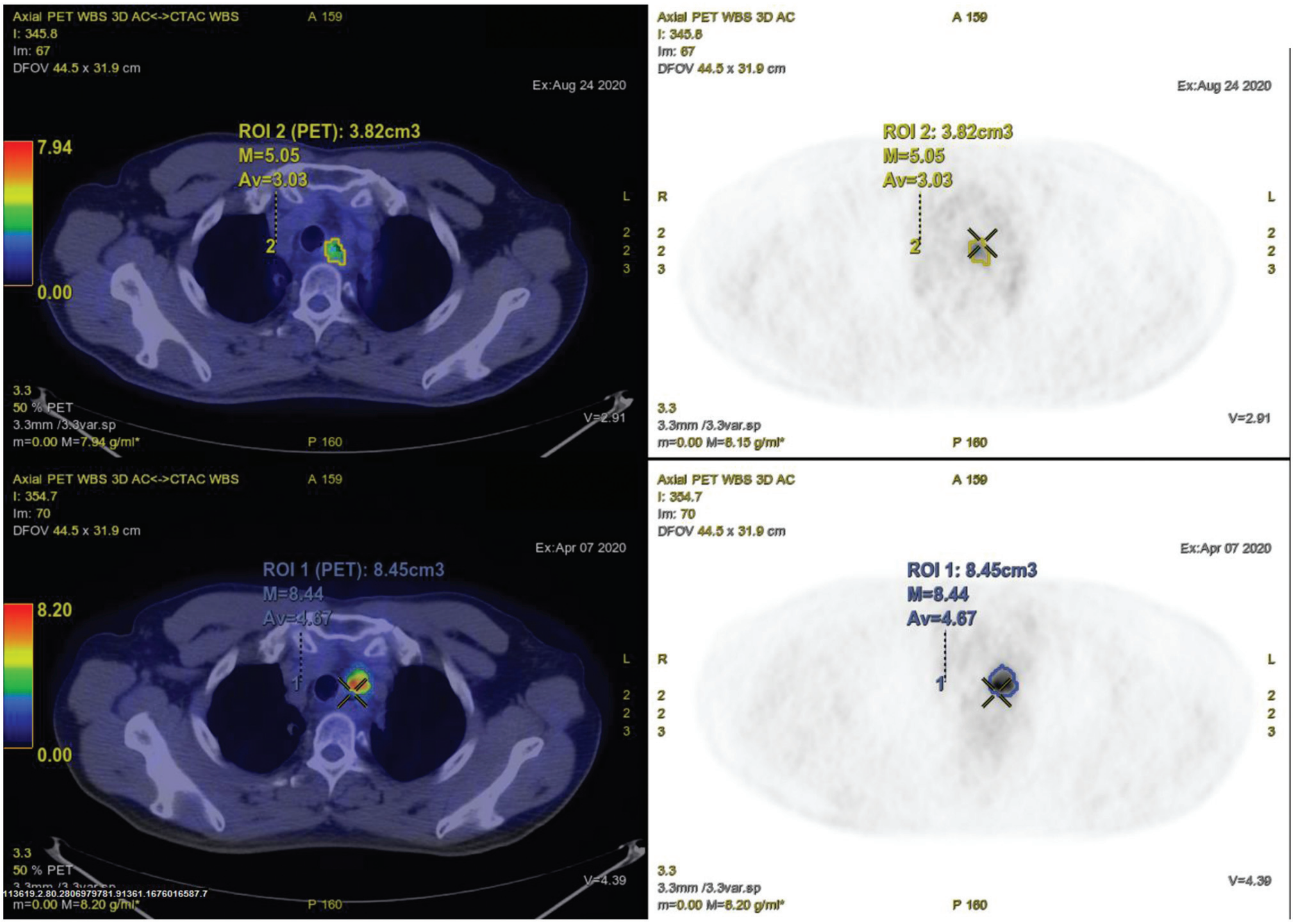


**Supplement Fig. 2** Sequential $^{18}$F-flouro-2-deoxy-glucose positron emission tomography/computed tomography (18F-FDG PET/CT) pre- and posttreatment. The male patient, 59 years old, with squamous cell esophageal cancer (cT3N1M0). *Lower row*: Pretreatment $^{18}$F-FDG PET/CT showed the ROI of upper third primary esophageal tumor (*crosshair*) with SUVmax: 8.44, SUVmean: 4.67, MTV: 8.45 ml. *Upper row*: posttreatment imaging was co-registered and sequential parameters of primary tumor (*crosshair*) are SUVmax: 5.05, SUVmean: 3.03, MTV: 3.82 ml. The free survival disease of the patient was 16 months until the last follow-up of the study.

 © 2023. The Author(s).
